# Psychiatry in the federal correctional system in Canada

**DOI:** 10.1192/bji.2020.56

**Published:** 2021-05

**Authors:** Colin Cameron, Najat Khalifa, Andrew Bickle, Hira Safdar, Tariq Hassan

**Affiliations:** 1 Correctional Service Canada/Government of Canada, FRCPC, National Senior Psychiatrist, Ottawa, Ontario, Canada; 2 MD, Associate Professor in Forensic Psychiatry, Queen's University Department of Psychiatry, Providence Care Hospital, Kingston, Ontario, Canada; 3 MRCPsych, Assistant Professor in Forensic Psychiatry, Queen's University Department of Psychiatry, Providence Care Hospital, Kingston, Ontario, Canada; 4 MBBS, Assistant Professor, Victoria Hospital, London Health Sciences Centre, London, Ontario, Canada; 5 FRCPC (Forensic Psychiatry), Divisional Chair and Clinical Director, Queen's University Department of Psychiatry, Providence Care Hospital, Kingston, Ontario, Canada. Email: hassant@providencecare.ca

**Keywords:** Corrections, prison, psychiatry, mental health, Canada

## Abstract

The unique challenges of the correctional healthcare environment are well-documented. Access to community-equivalent care, voluntary informed consent of offenders with mental disorder, violence risk, suicide risk, medication misuse, and clinical seclusion, confinement and segregation are just a few of the challenges faced by correctional psychiatric services. This paper shares experiences for dealing with the ongoing challenges for psychiatrists working in the field. It provides an overview of the current state of mental healthcare in the federal correctional system in Canada, the legislative framework and initiatives aimed at addressing the healthcare needs of federal inmates.

Correctional psychiatry is increasingly recognised as a subspecialty of forensic psychiatry, given the unique challenges of the prison healthcare environment. Criticisms abound about the apparent dual loyalties of correctional psychiatrists for both patient care and public safety. Access to community-equivalent care, voluntary informed consent of offenders with mental illness, violence and suicide risk, medication misuse and diversion, and clinical seclusion and restricted movement are just a few of the hot-button issues faced daily by correctional psychiatrists.^1,2^

Indeed, as in other jurisdictions, Correctional Service Canada (CSC) has come under increased scrutiny by prison advocacy groups and human rights lawyers. For example, there have been several litigations in recent years that have driven a number of reforms to the federal correctional system, including health services, many of which are outlined in this paper. Among these was the December 2017 decision by the Ontario Superior Court in *Corporation of the Canadian Civil Liberties Association v Her Majesty the Queen*, which ruled the lack of independent review of decisions to keep inmates in solitary confinement to be unconstitutional, and the court accepted evidence that this practice is harmful after as little as 48 h.^3^ Similarly, in January 2018 the British Columbia Supreme Court ruled in *British Columbia Civil Liberties Association v Canada* that sections of the Corrections and Conditional Release Act (CCRA) violate the Canadian Charter of Rights and Freedoms because it permits prolonged, indefinite solitary confinement, fails to provide independent review (external of CSC) of segregation placements and deprives inmates of the right to counsel at segregation review hearings.^4^ The court also ruled that it was violation of the Charter for mentally ill and disabled offenders to be placed in segregation, and opined that the practice disproportionately discriminates against Indigenous offenders.^4^ The court further suggested a 15-day maximum for segregation.^4^

Another example is the overrepresentation of Indigenous peoples in federal correctional facilities, which is a well-documented reality. In 2018–2019, Indigenous offenders represented 29% of the federally incarcerated population (compared with 4.9% of the total Canadian population).^5^ Compared with their non-Indigenous counterparts, Indigenous offenders are more likely to be incarcerated at a younger age, kept in custody longer, denied parole, overrepresented in segregation and assigned a high-risk offender designation.^5^ The Supreme Court of Canada tried to address this in 1999 in the case of *R v Gladue* when it ruled in favour of the appellant.^5,6^ This obliges sentencing judges to inquire into the adverse cultural factors that many Indigenous Canadians have endured. In a so-called Gladue analysis, these factors are noted, if present, in the personal history of the offender, which judges must take into consideration for sentencing, including looking at restorative justice remedies and all reasonable alternatives to incarceration.^5,6^ In spite of this, the disproportionate overrepresentation of Indigenous peoples in Canada's prisons persists, a fuller examination of which is beyond the scope of this paper.

It is in this challenging environment that correctional psychiatrists are working to develop the subspecialty.

## Canadian correctional system

Canadian adult prisons include two distinct systems: federal, responsible for offenders serving a sentence of 2 years or more; and provincial/territorial, responsible for remand inmates and those serving a sentence of under 2 years. In 2018–2019, there was an average of 38 786 adult offenders incarcerated in Canada on any given day, with 63% being in provincial/territorial custody and 37% in federal.^7^ In May 2020, the incarceration rate in Canada was 107/100 000 population, placing it near the middle of the pack among high-income nations (versus the USA 655, Israel 234, Australia 169, the UK 135, France 104, and Japan 39).^8^ The proportion of people in federal custody who are over 65 years is less (5.0%) than that of the general population in Canada, in which 16.1% are above this age.^9^ However, the federal prisoner population is becoming older. For instance, in 1993, only 0.9% of federal prisoners were >65 years.^5^ Moreover, in the same period the proportion of ‘older’ prisoners (over the age of 50 years) increased from 8.4 to 25.0%. This is consistent with worldwide trends thought to arise from ageing general populations, greater prosecution of sexual offences and historical offences, longer sentencing and tougher approaches to breach of supervision.^10^

As in other jurisdictions, the prevalence of mental disorders in federal Canadian prisons remains significantly higher than in the community, about 2–4 times higher for psychosis and major depression, and 10 times higher for antisocial personality disorder,^11^ with 70% of men and 79% of women having at least one DSM-IV-TR diagnosis.^12,13^

## Canadian federal correctional system

Correctional Service Canada (CSC) is responsible for the care, custody and rehabilitation of federally sentenced offenders. It is headed by a Commissioner who directly works with the Minister of Public Safety and Emergency Preparedness. There are eight sectors within CSC, of which the Health Services Sector is one.

In 2018–2019, CSC supervised approximately 23 000 offenders, about 60% in custody and 40% in the community. Women accounted for only 5.6% of federal offenders.^7^ CSC operates 43 institutions of various security classifications, including 5 women's institutions and 5 regional treatment centres.^14^ Additionally, CSC operates 14 community correctional centres for dangerous and long-term offenders and over 200 community residential facilities.^14^ CSC employs approximately 1200 healthcare staff, including nurses, psychologists, physicians, psychiatrists, social workers, occupational therapists and others. Most physicians, psychiatrists and dentists, however, are independent contractors rather than CSC employees. CSC also contracts with several community partners to provide healthcare services otherwise unavailable within CSC, including hospitals and laboratory services.

Additionally, CSC operates four Indigenous healing lodges, which are designed to provide culturally sensitive services and programmes to Indigenous peoples, incorporating Indigenous cultural and spiritual values and practices such as elder services, contact with nature and ceremonies.^15^ Healing lodges are intended to foster a sense of community cohesion and spiritual leadership, with the aim of addressing the criminogenic risk factors of inmates and community reintegration.^15^

The Corrections and Conditional Release Act (CCRA)^16^ mandates CSC to provide essential healthcare consistent with professionally accepted standards (section 86) and reasonable access to non-essential healthcare. It also stipulates that treatment must not be given to an inmate, or continued once started, without informed consent; and where an inmate lacks the capacity to consent, involuntary treatment must be governed by applicable provincial law (s. 88). Federal prisoners in Canada whose mental disorder warrants involuntary assessment or treatment are admitted to regional treatment centres, hybrid correctional mental health centres located in each of the five regions (Atlantic, Quebec, Ontario, Prairie and Pacific) that can use provincial mental health legislation for involuntary treatment (although the Regional Mental Health Centre in Quebec is not yet accredited as a hospital to be able to use the provincial mental health act). This is different from jurisdictions such as the UK, in which prisoners must be transferred out of the prison to community psychiatric hospitals in order to receive compulsory treatment. Discussion of the advantages and disadvantages of these alternative provisions is beyond the scope of this overview. In Canada, each province has its own mental health legislation and this governs detention for assessment and treatment within the federal correctional system. Therefore, the criteria for compulsory assessment and treatment of mental disorders varies somewhat between federal institutions located in different provinces.

## Amendments to the CCRA

In 2019, the CCRA underwent a significant revision, which made some 100 amendments.^17^ Principal among these was the abolition of administrative segregation and the introduction of a new model called structured intervention units (SIUs), which require that inmates have a minimum of 4 h out-of-cell time daily and 2 h of ‘meaningful’ human contact. The latter is supported by trained CSC officers to enhance offender participation in programmes and diversional activities.

The recent amendments to the CCRA also mandate in-person mental health assessments as soon as practicable and within 30 days of admission to CSC (s. 15.1 (2.01)). They also introduce mandatory daily healthcare assessments for all offenders in an SIU, and referral for a mental health assessment within 24 h of admission to an SIU (s. 37.1 (2)), which by policy must be carried out within 28 days. To implement these legislative changes, significant mental health staffing enhancements are planned which are to ramp up over 6 years and will result in 24/7 healthcare staffing in all five men's maximum security sites and all five women's institutions. It will also allow regional treatment centres to be staffed similarly to forensic psychiatric hospitals in the community. Additionally, the amendments introduced a formal patient advocacy service to help inmates to be aware of healthcare services within CSC and their rights and responsibilities with respect to these services, and to facilitate access to services. The amendments explicitly stipulate that CSC healthcare professionals are expected to advocate for their patients and provide patient-centred care.

The amendments also introduced independent external decision makers (IEDMs), who are to adjudicate when there are disputes between the institutional head and health services about continuing placement in an SIU or the need to modify the conditions of confinement. The decisions of IEDMs are binding. Additionally, IEDMs are expected to review all cases where an offender declines the requisite 4 h out-of-cell time for 5 days in any 15-day period and all offenders placed in an SIU for 90 days.

## The shift to independent health governance

There has been a progressive shift since 2007 towards independent health governance, and the healthcare system within CSC is externally accredited by Accreditation Canada. Since 2014, the line, functional and budget authorities have functioned independently from the rest of the CSC, allowing health services managers to determine healthcare staffing, policies and budget allocations. The Health Services Sector is led by the Assistant Commissioner of Health Services, who chairs the Health Services Executive Team (HSET) with input from the National Medical Advisory Committee (NMAC) and National Pharmacy and Therapeutics Committee.

The NMAC meets biannually to enhance physician engagement and leadership to develop the healthcare system, policies, guidelines, quality improvement, research and education. The by-laws of the NMAC are based on those typically seen in an academic health science centre or community healthcare organisation, with descriptions of roles and responsibilities for various positions, appointments, reporting authority, annual reviews, etc. There are five Regional Medical Advisory Committees with a similar mandate to engage physicians in each region.

## CSC mental health services

Mental health services in CSC are provided on a continuum from intake through to the conclusion of an offender's sentence, encompassing five integrated service domains: (a) mental health screening at intake; (b) primary mental healthcare; (c) intermediate mental healthcare; (d) psychiatric hospital care at regional treatment centres; and (e) clinical discharge planning and community mental health. Integrated mental health guidelines provide specifications for each domain, and interdisciplinary mental health teams coordinate the provision of mental health services across these domains.

Mental health indicators include rates of suicide and drug overdose. A CSC report looking at deaths in custody between 2000–2001 and 2015–2016 noted 154 suicides (18% of all deaths in custody), making suicide the most common cause of non-natural death, followed by 64 deaths by drug overdose (7% of all deaths in custody).^18^ Of note, suicide made up 64% of non-natural deaths in 2012–2013 versus only 39% in 2015–2016, and during this same time death by drug overdose had increased and become the most common cause of non-natural death in the Pacific Region.^18^ The service is tracking a number of mental health indicators prospectively (e.g. suicide, suicide attempts, non-suicidal self-injury, drug overdoses, changes in mental health needs, service utilisation) to inform clinical service delivery and policy and the impact of new services and policies.

## Recent CSC mental healthcare initiatives

Recently, CSC introduced several initiatives aimed at enhancing the quality of care. These include endorsing the Canadian Academy of Psychiatry and the Law (CAPL) guidelines on prescribing in correctional facilities,^19^ introducing guidance documents on the assessment and treatment of borderline personality disorder and attention-deficit hyperactivity disorder, and adopting a formal suicide prevention and intervention strategy.

The provision of addiction services has been enhanced through a revised guidance document on opioid agonist treatment, and the introduction of Self-Management and Recovery Training (SMART) programmes.^20^ Also, several institutions are now piloting overdose protection sites and prison needle exchange programmes.

Further, the ageing prison population brings additional challenges for correctional healthcare services. CSC has addressed this by developing a national policy framework that builds on current programmes and services to promote wellness and independence among its older persons in custody.^21^

Overarchingly, CSC is in the process of introducing the ‘patient medical home’ model, an evidence-based model in primary care that integrates health and mental health services under one umbrella to offer comprehensive care to patients through interdisciplinary healthcare teams and enhanced physician leadership.^22^

## Collaborations with academic institutions

In recent years, CSC has forged formal collaborations with academic institutions (Queen's University, Philippe-Pinel National Institute of Forensic Psychiatry, University of Saskatchewan and University of British Columbia) for psychiatric services, teaching and research. It is envisaged that such collaborations will improve the quality of care, enhance physician engagement and promote a culture of research and teaching.

## Challenges and future aspirations

The United Nations’ Nelson Mandela Rules outline the minimum recommended standards for the treatment of prisoners and stipulate that prisoners must have access to clinically independent community-equivalent healthcare provided with the patient's voluntary consent, in collaboration with community health services and with oversight from a competent public health regulator.^23^

Additionally, the World Health Organization's (WHO's) *Health in Prisons* guide challenges correctional authorities to provide prison healthcare that is integrated into a country's broader health services.^24^ Very few jurisdictions have met this challenge, exceptions being the UK, France, Norway and Australia. Canada's federal correctional system has yet to achieve this, although a shift has been made to independent health governance, and healthcare in provincial correctional facilities in British Columbia, Alberta, Quebec, Nova Scotia and Newfoundland are all now operated by the respective provincial health ministries.

Supported by a legislative framework, increased funding and enhanced physician leadership, CSC is going through a paradigm shift to meet the challenges set by the *Health in Prisons* guide^24^ to provide integrated and community-equivalent healthcare services. A number of initiatives have been rolled out or are in various stages of being rolled out which are intended to ensure that CSC meets this challenge and to address concerns raised by courts, civil liberties groups and prison advocacy groups.

The long-term impact of this paradigm shift to person-centred healthcare on patient outcomes and public safety has yet to be established and will be the source of ongoing evaluation.
